# Unexpected Functional Divergence of Bat Influenza Virus NS1 Proteins

**DOI:** 10.1128/JVI.02097-17

**Published:** 2018-02-12

**Authors:** Hannah L. Turkington, Mindaugas Juozapaitis, Nikos Tsolakos, Eugenia Corrales-Aguilar, Martin Schwemmle, Benjamin G. Hale

**Affiliations:** a Institute of Medical Virology, University of Zurich, Zurich, Switzerland; b Institute of Virology, Medical Center, University of Freiburg, Freiburg, Germany; c Faculty of Medicine, University of Freiburg, Freiburg, Germany; d Virology-CIET (Research Center for Tropical Diseases), Microbiology, University of Costa Rica, San José, Costa Rica; St. Jude Children's Research Hospital

**Keywords:** AKT signaling, RNA virus, bats, influenza, virulence factors

## Abstract

Recently, two influenza A virus (FLUAV) genomes were identified in Central and South American bats. These sequences exhibit notable divergence from classical FLUAV counterparts, and functionally, bat FLUAV glycoproteins lack canonical receptor binding and destroying activity. Nevertheless, other features that distinguish these viruses from classical FLUAVs have yet to be explored. Here, we studied the viral nonstructural protein NS1, a virulence factor that modulates host signaling to promote efficient propagation. Like all FLUAV NS1 proteins, bat FLUAV NS1s bind double-stranded RNA and act as interferon antagonists. Unexpectedly, we found that bat FLUAV NS1s are unique in being unable to bind host p85β, a regulatory subunit of the cellular metabolism-regulating enzyme, phosphoinositide 3-kinase (PI3K). Furthermore, neither bat FLUAV NS1 alone nor infection with a chimeric bat FLUAV efficiently activates Akt, a PI3K effector. Structure-guided mutagenesis revealed that the bat FLUAV NS1-p85β interaction can be reengineered (in a strain-specific manner) by changing two to four NS1 residues (96L, 99M, 100I, and 145T), thereby creating a hydrophobic patch. Notably, ameliorated p85β-binding is insufficient for bat FLUAV NS1 to activate PI3K, and a chimeric bat FLUAV expressing NS1 with engineered hydrophobic patch mutations exhibits cell-type-dependent, but species-independent, propagation phenotypes. We hypothesize that bat FLUAV hijacking of PI3K in the natural bat host has been selected against, perhaps because genes in this metabolic pathway were differentially shaped by evolution to suit the unique energy use strategies of this flying mammal. These data expand our understanding of the enigmatic functional divergence between bat FLUAVs and classical mammalian and avian FLUAVs.

**IMPORTANCE** The potential for novel influenza A viruses to establish infections in humans from animals is a source of continuous concern due to possible severe outbreaks or pandemics. The recent discovery of influenza A-like viruses in bats has raised questions over whether these entities could be a threat to humans. Understanding unique properties of the newly described bat influenza A-like viruses, such as their mechanisms to infect cells or how they manipulate host functions, is critical to assess their likelihood of causing disease. Here, we characterized the bat influenza A-like virus NS1 protein, a key virulence factor, and found unexpected functional divergence of this protein from counterparts in other influenza A viruses. Our study dissects the molecular changes required by bat influenza A-like virus NS1 to adopt classical influenza A virus properties and suggests consequences of bat influenza A-like virus infection, potential future evolutionary trajectories, and intriguing virus-host biology in bat species.

## INTRODUCTION

In 2012 and 2013, two novel influenza A virus (FLUAV) genomes were discovered by reverse transcription-PCR (RT-PCR) analysis of rectal samples from two species of Guatemalan and Peruvian fruit bats (1, 2). Due to high sequence and evolutionary divergence in multiple genomic segments compared to other FLUAVs, these viruses were designated the unique subtypes HL17NL10 and HL18NL11 (1–3). Functional characterization of bat FLUAVs and their encoded proteins using reverse genetic or reductionist techniques have since revealed remarkable properties of these viruses (reviewed in references 3 to 6). For example, bat FLUAV surface glycoproteins lack canonical receptor binding or destroying features (7–10), and these viruses preferentially enter polarized cells at the basolateral membrane (11). Thus, bat FLUAVs appear to have evolved unique mechanisms for entering host cells that are distinct from classical mammalian and avian FLUAVs. Other features that distinguish bat FLUAVs from other FLUAVs have yet to be explored in detail.

The precise host range and zoonotic potential of bat FLUAVs is unknown, although depending upon the experimental system used, these viruses can infect cells from an array of species, including bats, humans, and dogs (11–13). Reassortment of bat FLUAVs with classical FLUAVs has never been observed in experimental studies (14–16), although a cell-line derived from the bat species Pteropus alecto can support replication and reassortment of avian, swine, and human FLUAVs (17). In addition, there is evidence that bat species can be naturally infected with classical FLUAVs: in one study 30% of Ghanaian fruit bats analyzed were seropositive for avian H9 FLUAVs (18), and the respiratory and gastrointestinal tracts of North American little brown bats harbor receptors for both avian and mammalian FLUAVs (19). Thus, these data suggest that bats could represent an additional reservoir for FLUAVs, with the potential to provide either new gene segments or entire viruses that cross the species barrier with unknown consequences. Understanding fundamental properties of the newly described bat FLUAVs, such as their mechanisms to infect cells of certain species and how they modulate functions of the infected host cell environment, will be critical to assess their likelihood for causing disease in particular hosts.

We have focused on the nonstructural protein NS1 of bat FLUAVs, which shares only ∼50% sequence identity with other FLUAV NS1 proteins. NS1 is a multifunctional virulence factor that can contribute to virus host range and virulence (20, 21). A major role of NS1 during FLUAV infection is to antagonize the host innate interferon (IFN) system through multiple mechanisms, some of which appear to be strain dependent (22–31). We and others have recently demonstrated that bat FLUAV NS1 proteins, like classical FLUAVs, harbor a structurally and functionally conserved N-terminal double-stranded RNA-binding domain that is critical for potent IFN antagonism in human cells (15, 32, 33). The C-terminal effector domain (ED) of bat FLUAV NS1 may play a minor role in supporting IFN antagonism *in vitro* (15), possibly by promoting NS1 oligomerization (34–37). However, a striking finding is that deletion of the bat FLUAV NS1 ED has minimal impact on viral pathogenesis in a mouse model compared to deletion of this NS1 domain in a human H1N1 FLUAV (15). This suggests unappreciated functional differences between the EDs of bat and classical FLUAVs that remain to be explored. Here, we investigated one such activity of the NS1 ED: modulation of the host intracellular metabolic environment by binding the p85β regulatory subunit of phosphoinositide 3-kinase (PI3K) (38). The NS1 EDs of all classical human and avian FLUAV strains tested to date bind the inter-SH2 (iSH2) domain of host p85β to activate PI3K signaling (38–42). This activation generally benefits classical FLUAV replication, since viruses engineered to express NS1 proteins unable to bind p85β are attenuated for replication *in vitro* and for virulence *in vivo* (38, 43, 44). In this study, we found that bat FLUAV NS1 proteins uniquely lack the ability to bind host p85β and activate PI3K signaling. We map the bat FLUAV NS1 ED residues responsible for this atypical binding phenotype and characterize the impact that artificial introduction of this property into a model bat FLUAV has on virus propagation *in vitro*. Our data reveal an unexpected functional divergence of bat FLUAV NS1 proteins from classical FLUAV NS1 proteins and may suggest that specific metabolic conditions in the bat host *in vivo* negate any requirement for NS1 to evolve to engage with host PI3K signaling in these species.

## RESULTS

### Bat FLUAV NS1 proteins are unable to bind p85β.

**FIG 1:**
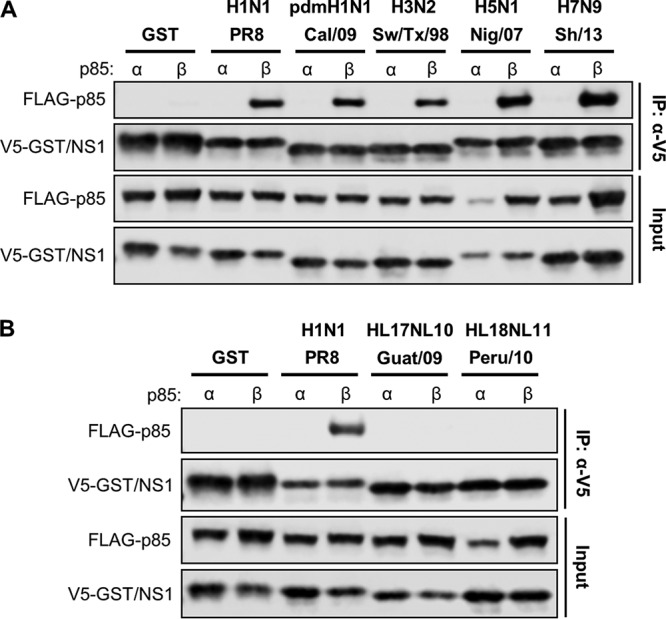
Strain-specific interaction of FLUAV NS1 proteins with human p85β. 293T cells were cotransfected for 48 h with plasmids expressing the indicated V5-tagged NS1 protein (or GST) and FLAG-tagged human p85α or p85β. After cell lysis, clarification, and anti-V5 immunoprecipitation, soluble (input) and pulldown (IP:α-V5) fractions were analyzed by SDS-PAGE and Western blotting. (A) Interaction studies of human (PR8, Cal/09), swine (Sw/Tx/98), and avian (Nig/07, Sh/13) FLUAV NS1 proteins. (B) Interaction studies of bat (Guat/09, Peru/10) FLUAV NS1 proteins. Data are representative of three independent experiments.

As part of a larger project to understand the interplay between FLUAV NS1 proteins and host PI3K, we screened a panel of different NS1s from various virus strains for their ability to interact with human p85β or the related isoform, p85α. To this end, 293T cells were cotransfected with plasmids expressing FLAG-tagged human p85α or p85β, together with plasmids expressing V5-tagged glutathione *S*-transferase (GST) or NS1 proteins from representative human, swine, avian, and bat strains. Soluble fractions were prepared from cell lysates at 48 h posttransfection and were subjected to immunoprecipitation with an antibody raised against the V5 tag. Western blot analysis of the resulting precipitates revealed that NS1 proteins from human (PR8, H1N1; and Cal/09, pdmH1N1), swine (Sw/Tx/98, H3N2), and avian (Nig/07, H5N1; and Sh/13, H7N9) strains were all capable of interacting specifically with human p85β, but not p85α (Fig. 1A). Surprisingly, NS1 proteins from bat FLUAVs (Guat/09, HL17NL10; and Peru/10, HL18NL11) were unable to precipitate either p85α or p85β (Fig. 1B). This phenotype has not previously been observed for a naturally occurring FLUAV NS1 protein but is akin to the more distantly related influenza B virus (FLUBV) NS1, which is also unable to bind PI3K subunits (45, 46).

### Bat FLUAV NS1 proteins do not show a species-specific interaction with p85β.

**FIG 2:**
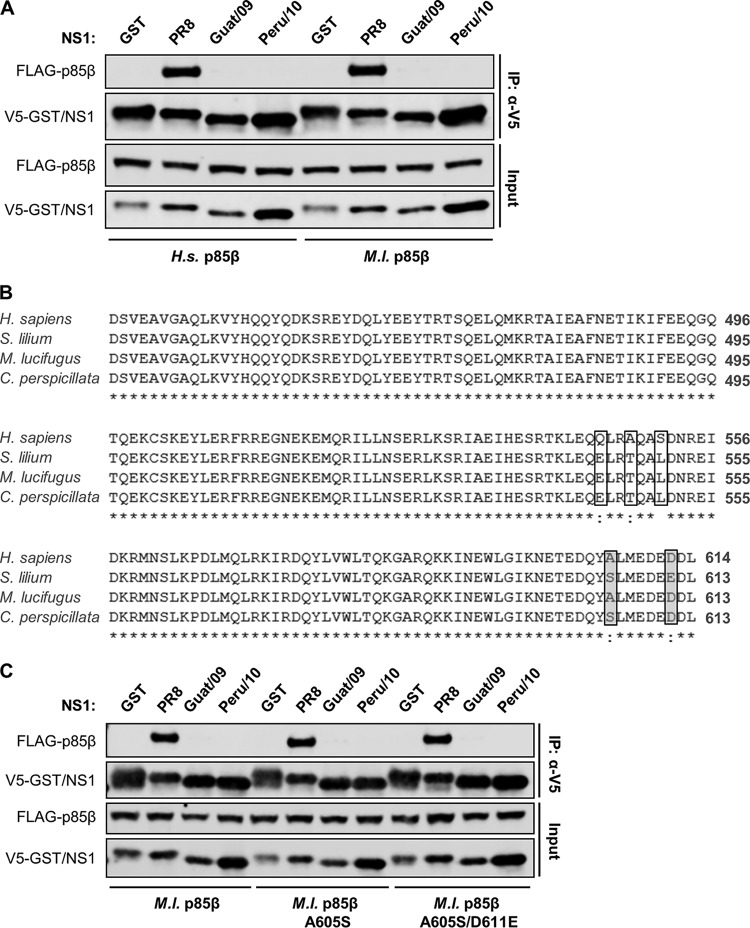
Bat FLUAV NS1 proteins do not show a species-specific interaction with p85β. (A) 293T cells were cotransfected for 48 h with plasmids expressing the indicated V5-tagged NS1 protein (or GST) and FLAG-tagged human (Homo sapiens, *H.s*.) or bat (Myotis lucifugus, *M.l*.) p85β. After cell lysis, clarification, and anti-V5 immunoprecipitation, soluble (input) and pulldown (IP:α-V5) fractions were analyzed by SDS-PAGE and Western blotting. (B) Sequence alignment of iSH2 domains from human and bat p85β. Human (H. sapiens), little yellow shouldered bat (S. lilium), little brown bat (M. lucifugus), and Seba's short-tailed bat (C. perspicillata) p85β iSH2 domain amino acid sequences (residues 496 to 614) were aligned. Human/bat differences are highlighted by boxes, and shading indicates residues differing between bat species. (C) Experiment performed as in panel A, except using the indicated bat p85β mutants. For panels A and C, the data are representative of three independent experiments.

To examine whether the interaction between bat FLUAV NS1 proteins and host p85β is species specific, we generated an expression vector for bat (Myotis lucifugus) p85β and assessed its coimmunoprecipitation with various NS1 proteins. Although PR8/NS1 interacted with this bat p85β to an extent similar to human p85β, neither the Guat/09 nor the Peru/10 bat FLUAV NS1 proteins were able to precipitate bat p85β (Fig. 2A). To exclude the possibility that this lack of p85β-binding was due to M. lucifugus not being the primary bat host species for bat FLUAVs, we sought to generate expression vectors mimicking p85β from the bat species Sturnia lilium (in which the HL17NL10 virus genome was first discovered [1]) and Carollia perspicillata (in which individual animals seropositive for HL18NL11 have been identified [2]). To this end, we cloned and sequenced the region encoding the p85β iSH2 domain from tissue originally derived from S. lilium and C. perspicillata. As shown in Fig. 2B, the human and bat p85β iSH2 domains are almost identical, with only five residue positions that differ. Three of these differences are conserved in the bat species sequenced (E544, T547, and L550; bat numbering), whereas two residue positions (605 and 611) differ between the M. lucifugus p85β iSH2 domain and the two other bat species (Fig. 2B). We therefore substituted these two residue positions in the M. lucifugus p85β for the respective residues from C. perspicillata (A605S) or S. lilium (A605S/D611E) and assessed their abilities to coprecipitate with NS1. However, neither bat p85β variant interacted with the bat FLUAV NS1 proteins, while their interaction with PR8/NS1 was unaffected (Fig. 2C). These data suggest that the bat FLUAV NS1 proteins have not specifically evolved to bind bat p85β.

### Bat FLUAV is an inefficient activator of PI3K signaling during infection.

**FIG 3:**
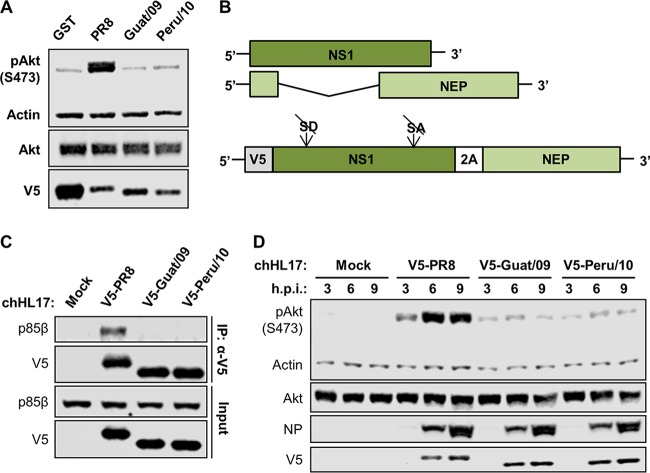
Bat FLUAV is an inefficient activator of PI3K signaling during infection. (A) Serum-starved 1321N1 cells stably expressing the indicated V5-tagged GST or NS1 proteins were lysed, and the levels of the indicated proteins were analyzed by SDS-PAGE and Western blotting. (B) Schematic representation of mRNAs generated by wild-type (upper) or engineered (lower) NS segments. SD, splice donor; SA, splice acceptor; 2A, 2A sequence from porcine teschovirus. (C) HAP-1 cells were infected for 8 h with chHL17 viruses expressing the indicated V5-tagged NS1 proteins at an MOI of 5 PFU/cell. After cell lysis, clarification, and anti-V5 immunoprecipitation, soluble (input) and pulldown (IP:α-V5) fractions were analyzed by SDS-PAGE and Western blotting. (D) Serum-starved HAP-1 cells were infected with chHL17 viruses expressing the indicated V5-tagged NS1 proteins at an MOI of 5 PFU/cell. Cells were lysed at 3, 6, and 9 h postinfection, and the levels of the indicated proteins were analyzed by SDS-PAGE and Western blotting. For panels A, C, and D, the data are representative of at least two independent experiments.

In contrast to PR8/NS1, and consistent with their inability to bind p85β, isolated expression of the V5-tagged NS1 proteins from Guat/09 or Peru/10 failed to stimulate phosphorylation of the downstream effector of PI3K, Akt (Fig. 3A). To assess further the impact of bat FLUAV infection on host PI3K signaling, we generated recombinant, chimeric bat FLUAVs possessing the six complete internal gene segments of Guat/09 (variant C3; chHL17) and modified HA and NA glycoprotein gene segments from PR8, where the untranslated regions were replaced by the genome ends and packaging sequences of the corresponding segments of Guat/09. Within these viruses, we also reengineered the NS segment to allow expression of different V5-tagged NS1 proteins (from PR8, Guat/09 or Peru/10) separated from Guat/09 nuclear export protein (NEP) by a 2A sequence (Fig. 3B). Immunoprecipitations from infected cell lysates revealed that endogenous p85β could be coprecipitated with V5-PR8/NS1 but not with the V5-tagged NS1 proteins derived from Guat/09 or Peru/10 (Fig. 3C). In addition, we observed that only the chimeric bat FLUAV expressing V5-tagged PR8/NS1 efficiently led to increased phosphorylation of Akt during infection (Fig. 3D). These infection data indicate that additional bat FLUAV internal gene segment products are unable to compensate bat FLUAV NS1 for either binding host p85β or activating PI3K.

### A small number of amino acid changes can establish an interaction between bat FLUAV NS1 and p85β.

**FIG 4:**
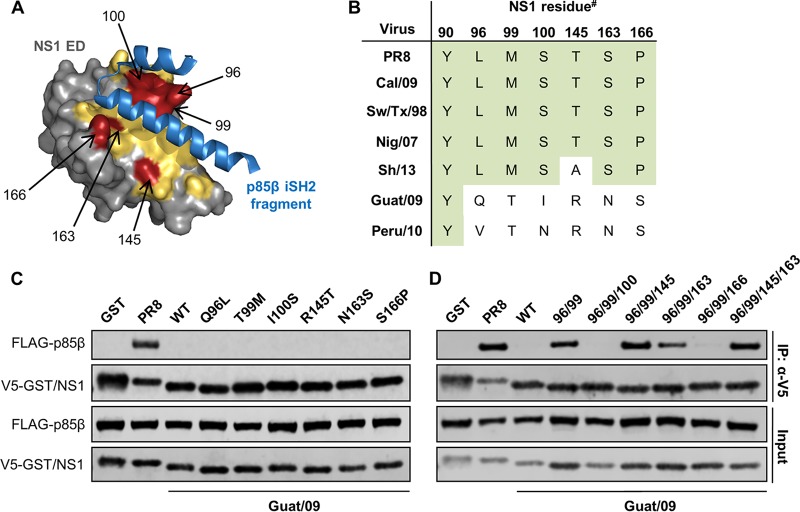
Engineering the bat FLUAV (Guat/09) NS1 protein to bind p85β. (A) Crystal structure of the PR8/NS1 ED in complex with part of the p85β iSH2 domain. The NS1 ED is shown in gray, with p85β contact residues highlighted in yellow. The p85β iSH2 domain is colored blue. The six NS1 amino acid residues chosen for mutagenesis studies (see panel B, Guat/09 numbering) are highlighted in red. The figure was generated using PyMOL with PDB 3L4Q. (B) Comparison of key NS1 ED residues between human (PR8, Cal09), swine (Sw/Tx/98), avian (Nig/07, Sh/13), and bat (Guat/09, Peru/10) NS1 proteins. #, Guat/09 numbering. (C and D) 293T cells were cotransfected for 48 h with plasmids expressing the indicated V5-tagged NS1 protein (or GST) and FLAG-tagged human p85β. After cell lysis, clarification, and anti-V5 immunoprecipitation, soluble (input) and pulldown (IP:α-V5) fractions were analyzed by SDS-PAGE and Western blotting. Data are representative of at least two independent experiments.

**FIG 5:**
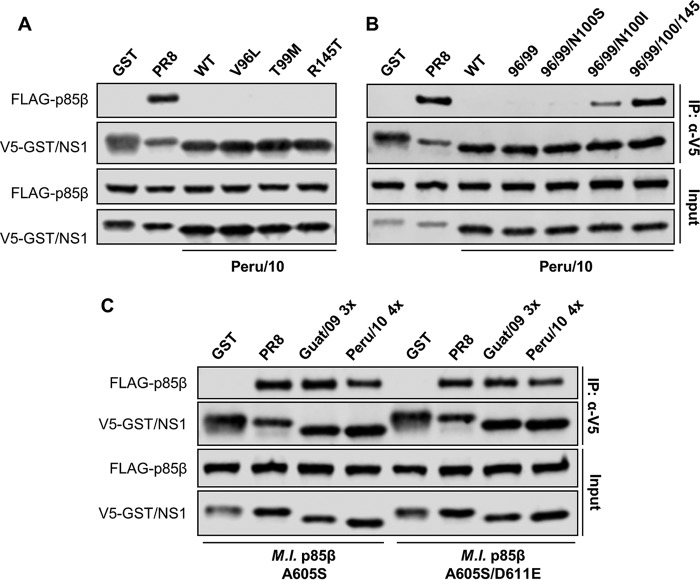
Engineering the bat FLUAV (Peru/10) NS1 protein to bind p85β. (A and B) 293T cells were cotransfected for 48 h with plasmids expressing the indicated V5-tagged NS1 protein (or GST) and FLAG-tagged human p85β. After cell lysis, clarification, and anti-V5 immunoprecipitation, soluble (input) and pulldown (IP:α-V5) fractions were analyzed by SDS-PAGE and Western blotting. (C) Experiment performed as in panel A, except using the indicated bat p85β mutants. Guat/09 3x = Q96L/T99M/R145T; Peru/10 4x = V96L/T99M/N100I/R145T. For all panels, data are representative of at least two independent experiments.

We analyzed the crystal structure of the PR8/NS1 ED in complex with the p85β iSH2 domain, taking into account recent mutagenesis data (Fig. 4A) (41, 47). Of the NS1 residues present at this interface, six were selected for substitution analysis in the bat FLUAV NS1 proteins based on their divergence from residues in NS1 proteins with the ability to bind p85β (Fig. 4A and B). Single amino acid substitutions at these six ED residues in Guat/09 NS1 (Q96L, T99M, I100S, R145T, N163S, and S166P; Guat/09 NS1 numbering) did not result in the establishment of human p85β binding (Fig. 4C). However, combining the amino acid substitutions Q96L and T99M resulted in partial p85β binding (Fig. 4D). The interaction between Guat/09 NS1 and human p85β could be enhanced further when the additional R145T substitution was introduced (Fig. 4D). Substitutions at other positions (particularly I100S) proved to be disruptive to the interaction (Fig. 4D). From these data, we infer that creation of a hydrophobic patch around positions 96, 99, and 100 in the Guat/09 NS1 ED is essential for determining the interaction with p85β. Largely similar, but distinct, results were obtained for substitution analysis of the Peru/10 NS1 protein: single amino acid substitution of V96L, T99M, or R145T, as well as double substitution of V96L and T99M, did not establish an interaction with human p85β (Fig. 5A and B). In fact, only when N100I was combined with double substitution of V96L and T99M could some weak p85β binding be observed, which was strongly enhanced by R145T (Fig. 5B). Notably, mutant Guat/09 and Peru/10 NS1 constructs generated to possess optimal p85β binding also exhibited the ability to interact with bat p85β (Fig. 5C).

### A chimeric bat FLUAV expressing an engineered p85β-binding NS1 protein exhibits cell-type-dependent, but species-independent, propagation phenotypes.

**FIG 6:**
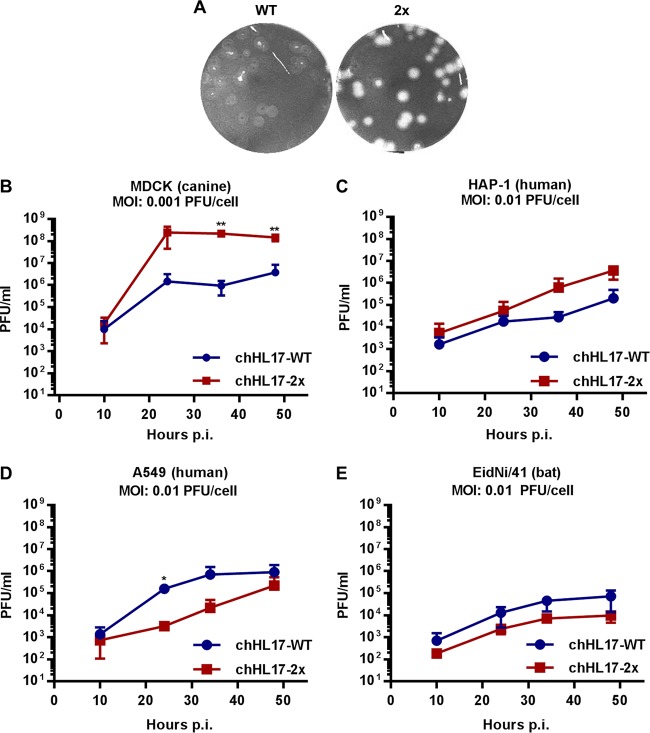
Characterization of a chimeric bat FLUAV expressing an engineered p85β-binding NS1 protein. (A and B) Plaque phenotype (A) and multicycle growth analysis (B) of chHL17-WT and chHL17-2x viruses in MDCK cells. (C to E) Multicycle growth analysis of chHL17-WT and chHL17-2x viruses in HAP-1 (C), A549 (D), and EidNi/41 (E) cells. For all growth curves, data represent mean values from three independent experiments ± the standard deviations. Significance was calculated using the Student *t* test comparing cHL17-WT and chHL17-2x virus titers at each time point (**, *P* < 0.01; *, *P* < 0.05).

Using reverse genetics, we generated chimeric bat FLUAVs as before (chHL17) but possessing authentic Guat/09 NS segments expressing either wild-type NS1 (WT) or NS1-Q96L/T99M (2x), which possesses p85β-binding ability with a minimal number of substitutions. Strikingly, we observed that chHL17-2x exhibited an altered plaque phenotype and markedly enhanced propagation capability (>100-fold-higher peak titers) in canine MDCK cells (Fig. 6A and B). Enhanced propagation of the chHL17-2x virus was also observed, to a lesser extent, in human HAP-1 cells (Fig. 6C). However, such a phenotype was not found in other cell types: the chHL17-2x virus exhibited slower replication kinetics in human A549 cells and a subtle attenuation in bat EidNi/41 cells (Fig. 6D and E). These data indicate that the Q96L/T99M substitutions in NS1 impact tissue culture replication of the chimeric bat FLUAV in a cell-type-dependent, but species-independent, manner.

### Role of p85β and PI3K activation in the phenotype of a chimeric bat FLUAV expressing NS1-Q96L/T99M.

**FIG 7:**
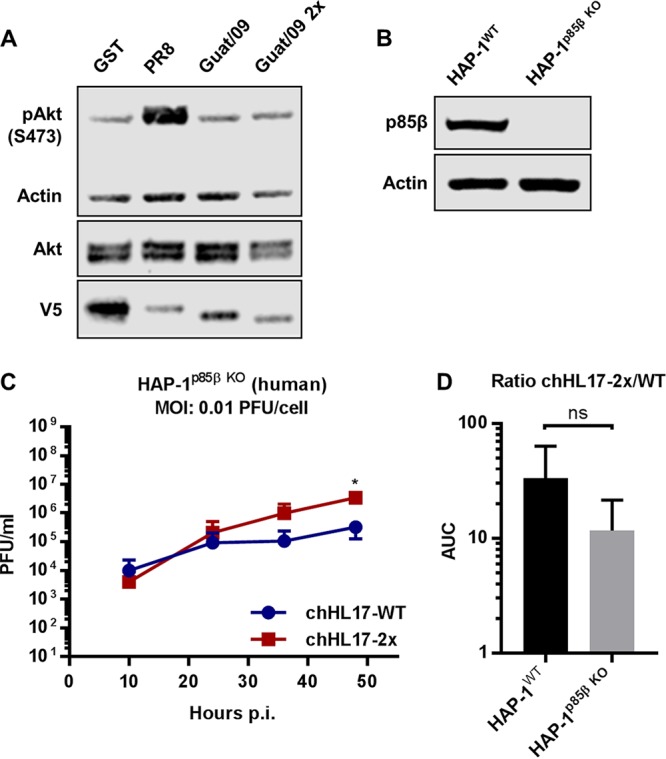
Role of p85β and PI3K activation in the phenotype of a chimeric bat FLUAV expressing NS1-Q96L/T99M. (A) Serum-starved 1321N1 cells stably expressing the indicated V5-tagged GST or NS1 proteins were lysed, and the levels of the indicated proteins were analyzed by SDS-PAGE and Western blotting. (B) Western blot analysis of wild-type or *PIK3R2* (p85β) knockout HAP-1 cells. For panels A and B, the data are representative of at least two independent experiments. (C) Multicycle growth analysis of chHL17-WT and chHL17-2x viruses in *PIK3R2* (p85β) knockout HAP-1 cells. Data represent mean values from three independent experiments ± the standard deviations. (D) Ratios of chHL17-2x to chHL17-WT propagation titers (represented as area under the curve [AUC] values) in wild-type and *PIK3R2* (p85β) knockout HAP-1 cells (original data from Fig. 6C and 7C). For panels C and D, the significance was calculated using the Student *t* test comparing chHL17-WT and chHL17-2x virus titers at each time point (*, *P* < 0.05) or the AUC ratios in each cell line (ns, nonsignificant).

We hypothesized that the engineered binding of Guat/09 NS1-Q96L/T99M to p85β, and potential activation of PI3K signaling, was responsible for modulating propagation of the chHL17-2x virus. However, similar to expression of the wild-type V5-tagged NS1 protein from Guat/09, expression of V5-Guat/09 NS1-Q96L/T99M failed to stimulate Akt phosphorylation (Fig. 7A), an observation supporting previous data that other NS1 determinants beyond efficient p85β binding can critically impact PI3K activation (41, 43, 48). In addition, we assessed propagation of the chHL17-2x virus in human HAP-1 cells that had been genetically engineered to lack p85β expression (Fig. 7B). Notably, the chHL17-2x virus also exhibited slightly enhanced propagation in this p85β knockout HAP-1 cell-line (Fig. 7C), which was similar to the enhancement previously observed in wild-type HAP-1 cells (Fig. 6C and 7D). Overall, these data suggest that, while the Q96L/T99M substitutions in Guat/09 NS1 permit host p85β-binding, they are not sufficient for this NS1 to activate host PI3K signaling. Furthermore, these NS1 substitutions may impact propagation of the chimeric bat FLUAV, at least in some host substrates, in a p85β/PI3K-independent manner.

## DISCUSSION

Functional characterization of proteins encoded by the novel bat FLUAV sequences has so far revealed some remarkable properties, most notably that the HA-like protein does not bind canonical FLUAV sialic acid receptors, and the NA-like protein lacks neuraminidase activity (7–10). Here, we describe an additional feature of classical mammalian and avian FLUAVs that is missing in their bat-derived counterparts: the multifunctional bat FLUAV NS1 virulence factor is unable to bind host p85β or activate PI3K signaling. This property seems important for the efficient replication of many classical FLUAV strains in tissue culture and *in vivo* (38, 43, 44, 47). Using a structure-guided approach, we found that p85β binding could be engineered into the bat FLUAV NS1 proteins with a minimum of only two amino acid substitutions (Q96L/T99M) and that a chimeric bat FLUAV expressing NS1 with these substitutions had improved propagation kinetics in some tissue culture systems (canine MDCK and human HAP-1) but a seemingly attenuated phenotype in others (human A549 and bat EidNi/41). Notably, this disparate impact did not correlate with host species, since two different human cell lines were identified in which the outcome of infection was opposite. Therefore, cell type (and thus unknown differential gene/protein expression profiles or polymorphisms) likely determine the phenotypic result. Interestingly, the propagation phenotype in human HAP-1 cells of the chimeric bat FLUAV expressing NS1-Q96L/T99M was not diminished in an isogenic cell line completely lacking p85β expression, suggesting that the amino acid substitutions used to permit p85β binding by NS1 also modify an additional property of this multifunctional viral protein that has yet to be identified. This will certainly be an interesting area to explore in the future and may impact our understanding of other classical influenza A viruses and the biological consequences of certain NS1 polymorphisms that they harbor. Such observations will also have implications when assessing potential virological consequences of the p85β-binding property arising naturally in bat FLUAVs.

Engineering p85β binding into the bat FLUAV NS1 protein did not result in detectable activation of PI3K signaling, indicating that additional regions of NS1 are essential for this function. Indeed, while p85β binding is necessary for NS1-mediated PI3K activation (38), our data suggest that it is not sufficient, an observation consistent with previous work (41, 48). Glutamic acid residues at human FLUAV NS1 positions 96 and 97 (equivalent to bat FLUAV NS1 positions 97 and 98) do not interact directly with p85β, but have been proposed to form part of an “activating interface” with the p110 catalytic subunit of the p85β-containing PI3K heterodimer (41). Although these are the only non-p85β-interface residues so far implicated in NS1-activated PI3K signaling, they are conserved in the bat FLUAV NS1 proteins, meaning that another unidentified region of classical FLUAV NS1 proteins must be responsible for ensuring PI3K activation. Experiments to uncover this specific region are under way, but it is therefore apparent that more than the two to four amino acid substitutions required for p85β binding would be necessary to allow bat FLUAV NS1 proteins to fully acquire PI3K activation potential, constituting a relatively high genetic barrier to natural evolution of this property.

Our studies suggest that interactions between NS1 and p85β are not species specific: bat FLUAV NS1 proteins do not bind bat p85β. Thus, an open question is why bat FLUAVs have not retained (or evolved) this property like classical mammalian and avian FLUAVs? One hypothesis for this observation is that the natural host environment encountered *in vivo* by bat FLUAVs is in a particular metabolic state such that viral augmentation of PI3K signaling would not alter host physiology. Indeed, recent genome sequencing and analysis studies have begun to uncover major differences between bats and other species and have linked specialized adaptations of bats (e.g., flying ability, longevity, hibernation, and echolocation) with specific adaptive evolution of energy metabolism genes. Such genes include those involved in oxidative phosphorylation (49), insulin/growth factor receptor signaling (50), and DNA damage repair and apoptosis (51), pathways otherwise known to be regulated by PI3K in many mammalian systems (52, 53). Possible links between positive selection in bat metabolic genes and decreased sensitivity to PI3K-mediated regulation need to be validated experimentally but could account for bat FLUAV NS1 proteins adopting a different evolutionary path to those from their classical FLUAV counterparts in other species. Such functional divergence of bat FLUAV NS1 proteins underscores the uniqueness of these newly discovered viruses and suggests new and intriguing virus-host interaction biology in bat species that remains to be explored.

## MATERIALS AND METHODS

### Cells.

MDCK (canine), 293T (human), A549 (human), 1321N1 (human), EidNi/41 (bat; Eidolon helvum) (54), and CarperAEC.B-3 (bat; Carollia perspicillata) (55) cells were cultured in Dulbecco's modified Eagle's medium (DMEM) supplemented with 10% fetal bovine serum (FBS), 100 U/ml penicillin, and 100 μg/ml streptomycin (Gibco Life Technologies). HAP-1 cells (Horizon Discovery, Austria) were cultured in Iscove's modified Dulbecco's medium (IMDM) supplemented with the same additives, as well as 200 μM GlutaMAX (Gibco Life Technologies). The HAP-1 cell line engineered by CRISPR/Cas9 to have a 2-bp deletion in the *PIK3R2* gene (encoding p85β) was purchased from Horizon Discovery (catalog no. HZGHC003292c006). All cells were maintained at 37°C with 5% CO_2_.

### Expression plasmids.

The NS1 cDNA sequences from A/Swine/Texas/4199-2/98 (Sw/Tx/98, H3N2), A/Nigeria/OG10/2007 (Nig/07, H5N1), A/Shanghai/2/S1078/2013 (Sh/13, H7N9), A/little yellow shouldered bat/Guatemala/153/2009 (Guat/09, HL17NL10), and A/flat-faced bat/Peru/033/2010 (Peru/10, HL18NL11) were PCR amplified from existing plasmids and ligated in frame with an N-terminal V5-tag into modified pLVX-IRES-ZsGreen1 or pLVX-IRES-Puro plasmids (Clontech, USA). Similar plasmids expressing GST and NS1 sequences from A/Puerto Rico/8/1934 (PR8, H1N1) or A/California/04/09 (Cal09, pdmH1N1) have been described previously (47). All NS1-encoding cDNAs contained silent mutations in the splice acceptor site to prevent the expression of NEP. Human p85α and p85β cDNA sequences from existing plasmids or the bat (Myotis lucifugus) p85β cDNA sequence generated using the GeneArt gene synthesis service (Thermo Fisher) were PCR amplified and ligated in frame into p3xFLAG-CMV-7.1 (Sigma-Aldrich) so as to express with N-terminal FLAG tags. Two-step overlap PCR or QuikChange II XL (Agilent Technologies, Switzerland) was used to introduce site-directed mutations into cDNAs as required. The identity of all constructs was confirmed by sequencing. All plasmid transfections were performed using Fugene HD (Promega) at a 1:3 DNA/transfection reagent ratio. As appropriate, generation of lentiviruses and the production of stable cell lines constitutively expressing proteins of interest were carried out as previously described (38).

### Virus reverse genetics.

Plasmids for rescuing chimeric, recombinant HL17NL10 virus (variant C3: PA-S550R and M2-N31S/T70A) were described previously (14). pHW2000 rescue plasmids comprising the genome ends and packaging sequences of HL17NL10 and the PR8 HA or NA glycoprotein open reading frames (ORFs) were generated as described previously (15). Briefly, the untranslated regions of PR8 HA and NA genome segments were replaced with the genome ends and packaging sequences of the corresponding segments of HL17NL10 by assembly PCR. Plasmids encoding NS segments where NS1 and NEP ORFs were separated by the 2A self-cleaving peptide of porcine teschovirus (allowing N-terminal V5-tagging of NS1) were generated by assembly PCR according to a previous strategy (56). All generated NS chimeras contained silent mutations in the splice acceptor and donor sites of the ORFs to prevent aberrant splicing, and duplicated genome packaging sequences were introduced flanking the NS1 ORF to avoid packaging defects. QuikChange II XL was used to introduce site-directed mutations into cDNAs as required. For rescues, 6 × 10^5^ 293T cells were seeded in six-well plates and cotransfected 24 h later with the eight pDZ- or pHW2000-based plasmids. At 24 h posttransfection, the cells were washed once in sterile phosphate-buffered saline (PBS) and 3 × 10^5^ MDCK cells were added in DMEM supplemented with 1 μg/ml TPCK (tosylsulfonyl phenylalanyl chloromethyl ketone)-treated trypsin (Sigma-Aldrich, St. Louis, MO). Forty-eight hours later, the supernatants were harvested, the viruses were plaque purified, and virus stocks were grown and titrated according to standard methods in MDCK cells. RNA was extracted from stock aliquots using a ReliaPrep RNA Cell miniprep system (Promega), and the NS genomic segments of each virus were fully sequenced after segment-specific RT-PCR to ensure absence of undesired mutations.

### Virus infections.

For all infections, viruses were diluted in PBS supplemented with 100 U/ml penicillin, 100 μg/ml streptomycin (Gibco Life Technologies), 0.3% bovine serum albumin (Sigma-Aldrich), and 1 mM Ca^2+^/Mg^2+^. For HAP-1 cells, cells were seeded onto poly-l-lysine (Sigma-Aldrich)-coated plates and infected 24 h later at the indicated multiplicity of infection (MOI) for 1 h at 37°C. After infection, the cells were washed three times and overlaid with FBS-free IMDM. For serum starvation experiments, the cells were washed three times with FBS-free IMDM and incubated overnight prior to infection.

For virus growth analyses, cells in 12-well plates were infected with the indicated virus at the indicated MOI. After infection at 37°C for 1 h, the cells were washed three times with PBS and then overlaid with DMEM supplemented with 100 U/ml penicillin, 100 μg/ml streptomycin (Gibco Life Technologies), and 0.5 μg/ml TPCK-treated trypsin (Sigma-Aldrich). Supernatant samples were harvested at the indicated time points and stored at −80°C prior to titration by standard plaque assay.

### Sequencing of p85β (*PIK3R2*) iSH2 domains from bat species.

Bats (S. lilium) were captured with mist nets. Specimens were collected and processed after approval of the Institutional Committee of Care and Use of Animals of the University of Costa Rica (CICUA-36-13) according to national guidelines for animal caring described in the Costa Rica National Law for Animal Welfare 7451. For sequencing, 2.5 × 10^5^ CarperAEC.B-3 cells seeded in 12-well plates, or an equivalent amount of cellular material from S. lilium, were lysed and RNA extracted using a ReliaPrep RNA cell miniprep system (Promega). RT-PCR products were generated and directly sequenced using specific primers annealing to conserved regions of *PIK3R2* flanking the sequence encoding the p85β iSH2 domain.

### Immunoprecipitations.

Transfected (2 μg total DNA, 48 h in 293T) or infected (MOI of 5 PFU/cell, 8 h in HAP-1) cells in 25-cm^2^ flasks or six-well plates were lysed on ice in 1 ml of 20 mM Tris-HCl (pH 7.8), 5 mM EDTA, 0.5% (vol/vol) NP-40, and 650 mM NaCl, supplemented with a cOmplete mini protease inhibitor tablet (Roche, Switzerland). Lysates were clarified using a 29G (0.33-mm) needle and centrifugation at 13,000 rpm for 40 min at 4°C. Soluble fractions were then incubated with 1 μg of anti-V5 antibody (Bio-Rad) for 2 h at 4°C prior to further incubation overnight at 4°C with 12.5 μl protein G-Sepharose beads (Sigma-Aldrich). After extensive washing, the remaining proteins were dissociated from the beads using urea disruption buffer (6 M urea, 1 M β-mercaptoethanol, 4% sodium dodecyl sulfate [SDS]) and heating at 95°C for 10 min. Samples were stored at −20°C until analysis by SDS-PAGE and Western blotting.

### Akt phosphorylation assays.

1321N1 cells stably expressing the indicated V5-tagged protein were seeded into 12-well plates and serum starved the following day for 1 h. Cell lysates were harvested in urea disruption buffer and stored at −20°C until analysis by SDS-PAGE and Western blotting.

### SDS-PAGE and Western blotting.

Samples were sonicated to shear nucleic acids and then boiled for 5 min. Polypeptides were resolved by SDS-PAGE on NuPAGE 4-12% Bis-Tris protein gels (Thermo Fisher), followed by transfer to nitrocellulose membranes (GE Healthcare Life Sciences). Proteins were detected by Western blotting with the following primary antibodies: mouse anti-V5 (Bio-Rad, MCA1360), mouse anti-FLAG (Sigma-Aldrich, F3165), mouse anti-p85β (T15; AbD Serotech, MCA1170G), rabbit anti-actin (Sigma-Aldrich, A2103), rabbit anti-Akt (Cell Signaling Technology, catalog no. 4691), rabbit anti-pAkt (S473; Cell Signaling Technology, catalog no. 4060), rabbit anti-NS1 (1-73) (57) (kindly provided by Adolfo García-Sastre, Icahn School of Medicine at Mount Sinai, New York, NY), and rabbit anti-NP (kindly provided by Silke Stertz, University of Zurich, Zurich, Switzerland). Secondary antibodies were fluorochrome-conjugated: anti-mouse (ThermoFisher Scientific, SA5-10176) and anti-rabbit (Thermo Fisher Scientific, SA5-10036). A LI-COR Odyssey Fc scanner was used for detection.

### Structural analyses.

Structural representations were visualized using the appropriate PDB file and PyMOL (58).
